# Physicochemical, Antioxidant, Microstructural Properties and Bioaccessibility of Dark Chocolate with Plant Extracts

**DOI:** 10.3390/molecules26185523

**Published:** 2021-09-11

**Authors:** Szymon Poliński, Sylwia Kowalska, Patrycja Topka, Aleksandra Szydłowska-Czerniak

**Affiliations:** 1 Department of Analytical Chemistry and Applied Spectroscopy, Faculty of Chemistry, Nicolaus Copernicus University in Toruń, Gagarina 7, 87-100 Toruń, Poland; szymon.polinski@kopernik.com.pl (S.P.); skowalska@umk.pl (S.K.); 2 Fabryka Cukiernicza Kopernik S.A., Stanisława Żółkiewskiego 34, 87-100 Toruń, Poland; patrycja.topka@wp.pl

**Keywords:** dark chocolate, plant extracts, antioxidant capacity, phenolics content, in vitro digestion, scanning electron microscopy with dispersive energy spectroscopy

## Abstract

In this study, dark chocolates (DCh) containing zinc lactate (ZnL) were enriched with extracts from elderberries (EFrE), elderflowers (EFlE), and chokeberries (ChFrE) to improve their functional properties. Both dried plant extracts and chocolates were analyzed for antioxidant capacity (AC) using four different analytical methods: 2,2-diphenyl-1-picrylhydrazyl (DPPH), 2,2′-azino-bis(3-ethylbenzothiazoline-6-sulfonic acid) (ABTS), cupric ion-reducing antioxidant capacity (CUPRAC), and ferric-reducing antioxidant power (FRAP), while total phenolic content (TPC) was determined by Folin–Ciocalteu (F–C) assay. An increase in antioxidant properties of fortified chocolates was found, and the bioaccessibility of their antioxidants was evaluated. The highest AC and TPC were found in ChFrE and chocolate with chokeberries (DCh + ChFrE) before and after simulated in vitro digestion. Bioaccessibility studies indicated that during the simulated digestion the AC of all chocolates reduced significantly, whereas insignificant differences in TPC results were observed between chemical and physiological extracts. Moreover, the influence of plant extracts on physicochemical parameters such as moisture content (MC), fat content (FC), and viscosity of chocolates was estimated. Furthermore, scanning electron microscopy with dispersive energy spectroscopy (SEM-EDS) was used to analyze surface properties and differences in the chemical composition of chocolates without and with additives.

## 1. Introduction

Dark chocolate (DCh) can be part of a diet that affects global public health and meets current dietary recommendations due to the antioxidant properties of cocoa mass, a principal ingredient containing high amounts of polyphenols, flavonoids, vitamins, and minerals [1].

On the other hand, the characteristics and manufacturing of chocolate potentially allow the addition of pro-health ingredients such as dried fruits and other parts of plants. Therefore, in recent years, the effects of dried fruits and plants, such as prunes, papaya, apricots, raisins, cranberries, lychee, longan, nettle, red raspberry leaves, Sakura green tea, turmeric powder, and yellow tea powdered extracts on the antioxidant capacity (AC) of white, milk, semisweet, and dark chocolates determined by 2,2-diphenyl-1-picrylhydrazyl (DPPH), 2,2′-azino-bis(3-ethylbenzothiazoline-6-sulphonic acid) (ABTS), ferric-reducing antioxidant power (FRAP), and oxygen radical absorbance capacity (ORAC), as well as levels of dominant antioxidants have been investigated [2,3,4,5,6,7]. The incorporation of dried fruit and plant extracts into chocolates influenced their antioxidant and sensory properties and contributed to the dietary intake of polyphenolic antioxidants.

It is worth noting that there has been no reference to the changes in the antioxidant potential of chocolates fortified with elderberry and chokeberry extracts. However, black chokeberry (*Aronia melanocarpa*) fruits are some of the richest sources of bioactive compounds, including flavonols, flavanols, phenolic acids, proanthocyanidins, and anthocyanins. Although these fruits are rarely used for direct consumption due to a tart and bitter taste caused by a high content of phenolic compounds, they can be widely utilized for the production of natural powders and dietary supplements with health-promoting properties [8]. Moreover, the black fruits, flowers, leaves, and bark of elderberry (*Sambucus nigra* L) contain high amounts of bioactive compounds such as phenolics, anthocyanins, and others possessing strong antioxidant, antibacterial, antiviral, antidepressant, antitumor, anti-inflammatory, antihypoglycemic, immune-modulating properties as well as the ability to reduce body fat and lipid concentration [9,10]. Nevertheless, gastrointestinal digestion affected elderberry antioxidants causing changes in their AC, chemical structure, and stability [11].

On the other hand, in vitro digestion models are becoming useful tools for studying the digestive properties of chocolates and cocoa-based products and for understanding the mechanisms of lipid absorption and the bioaccessibility of amino acids, bioactive amines, polyphenols, and other antioxidants [6,12,13].

Taking into account the health-promoting properties of DCh, elderberries, elderflowers, and chokeberries, it is possible to put forward the hypothesis that chocolates fortified with these dried plants and zinc lactate (ZnL) could be products for the enhancement of consumers’ health.

Therefore, this work aimed to quantify for the first time the AC of DCh with ZnL after supplementation of powdered extracts from elderberries (EFrE), elderflowers (EFlE), and chokeberries (ChFrE). Moreover, the stability of their AC and total phenolic content (TPC) after in vitro gastrointestinal digestion using the DPPH, ABTS, cupric reducing antioxidant capacity (CUPRAC), FRAP, and Folin–Ciocalteu (F–C) methods, respectively, was estimated and discussed. The influence of these dried fruits and flowers on the physicochemical properties of DCh was determined. Finally, scanning electron microscopy (SEM) images of the supplemented chocolates and SEM with dispersive energy spectroscopy (EDS) techniques were used to study changes in particle shapes after fortification with nutritional additives rich in bioactive components, and to evaluate the chemical composition of the prepared chocolate samples.

## 2. Results and Discussion

### 2.1. Antioxidant Capacity of Plant Extracts

In order to receive reliable data on the overall antioxidant potential of EFrE, EFlE, and ChFrE, two radical scavenging assays (DPPH and ABTS) and two reducing methods (CUPRAC and FRAP) were applied. The obtained AC results (Table 1) revealed the same trend in the ability of the investigated extracts to act against free radicals (DPPH^•^ and ABTS^•+^) as the capability of compounds present in them to change the oxidation state of transition metals in complexes (copper(II)-neocuproine and iron(III)-2,4,6-tripyridyl-s-triazine).

Results of AC and TPC illustrated that ChFrE was the richest source of hydrophilic and lipophilic antioxidants (Table 1). Therefore, the Duncan test indicated that AC and TPC in ChFrE were significantly higher than DPPH, ABTS, CUPRAC, FRAP, and TPC in EFrE and EFlE. On the other hand, differences in AC of the same extract determined by four modified analytical methods were observed. These discrepancies between the AC results may be attributed to the different mechanisms of the applied analytical methods, including DPPH and ABTS mixed-mode methods involving both electron transfer (ET) and hydrogen atom transfer (HAT) mechanisms, as well as ET assays such as CUPRAC and FRAP. Simultaneous determination of lipophilic and hydrophilic antioxidants in various matrices can be achieved using CUPRAC and ABTS methods, while the DPPH test is suitable for evaluating the AC of lipophilic compounds. In contrast to the DPPH procedure, FRAP assay is specific for potential hydrophilic antioxidants, but it does not respond well to lipophilic antioxidants [14]. For this reason, AC results of water extracts from EFrE, EFlE, and ChFrE revealed higher reducing potencies for CUPRAC assay and antiradical activity determined by the ABTS method than scavenging capacity of DPPH radical and reducing abilities analyzed by the FRAP test (Table 1).

For comparison, water extract from chokeberries also possessed high antioxidant properties, and its AC when analyzed by the same antioxidant assays showed a similar tendency: ABTS (219.3 μmol TE/g) ≈ CUPRAC (212.9 μmol TE/g) > DPPH (87.2 μmol TE/g) > FRAP (57.4 μmol TE/g) [15].

It is noteworthy that the EFrE had significantly lower radical scavenging properties of DPPH^•^ and ABTS^•+^ (11 times lower) as well as reducing abilities of Fe(III) (10 times lower) and Cu(II) (20 times lower) ions than ChFrE. However, DPPH, ABTS, FRAP, and TPC values in EFlE were above 2 times higher than those found for EFrE. Similarly, EFlE showed significantly higher reducing power determined by the CUPRAC method than EFrE (Duncan test, Table 1).

The AC and TPC data for ChFrE and EFrE are in agreement with those reported by other studies [16,17,18], showing that black chokeberries had much higher radical-scavenging activities evaluated by the DPPH and ABTS tests and higher amounts of phenolics than elderberries (Table 2). 

Additionally, the flower alcoholic extracts from elderberries exhibited stronger neutralizing activity of DPPH^•^ and ABTS^•+^ radicals and higher TPC in comparison with those results for berry extracts (Table 2 [19,20]). This suggests that cinnamic acids, flavonols, and anthocyanins are dominant in elderberry flowers. It is evident that the ABTS and TPC values obtained by Mikulic-Petkovsek et al. [20] were significantly lower than those determined for our EFrE and EFlE samples (Table 1 and Table 2). On the contrary, Młynarczyk et al. [21] found a somewhat higher ABTS for elderberries than ABTS for elderflowers from cultivars grown in the wild and in an orchard. However, elderflowers were richer in TPC when compared with phenolic levels in fruits (Table 2).

### 2.2. Antioxidant Capacity and Total Phenol Content in Chocolates before and after In Vitro Simulated Digestion

Regarding the antioxidative characteristics of the investigated DCh enriched with EFrE, EFlE, and ChFrE, the AC determined by four different analytical methods and TPC results followed the same trend as for plant extracts.

It can be noted that the addition of plant extracts to DCh with ZnL caused a plant extract type-dependent statistically significant increase in DPPH, ABTS, CUPRAC, FRAP, and TPC results of fortified chocolate samples (Duncan test, Table 3). 

Therefore, DCh + ChFrE was the richest source of antioxidants and revealed the highest AC and TPC, whereas DCh without plant extracts had the lowest antioxidant properties (Table 3). It is evident that enrichment of DCh with EFrE containing the lowest amounts of antioxidants increased the ABTS and FRAP by about 50% and CUPRAC above 4%, but insignificant differences for DPPH and TPC values were observed between DCh and DCh + EFrE (Duncan test, Table 3). This fact confirms that cocoa and its derivatives as the main ingredients of DCh are renowned sources of natural phenolic compounds such as flavanols (epicatechin, catechin), proanthocyanidins, and anthocyanins, which have antioxidant properties. Additionally, DCh can contain other well-known antioxidants such as Maillard reaction products generated during high-temperature processes: drying, roasting, and conching [22].

For comparison, the addition of various plant extracts such as red raspberry leaves [5], yellow tea [7], Sakura green tea, turmeric powder [6], black carrot [12], dried cranberries, and prunes [2] to DCh samples and chocolate products caused an increase in AC and TPC analyzed by DPPH, ABTS, FRAP, and F–C assays (Table 4). Unexpectedly, the DPPH, ABTS, and TPC results [3] for all chocolate pralines produced with the addition of either longan or lychee were significantly lower than the antioxidant properties of control samples (Table 4). The authors explained that replacing chocolate corpus with a filling containing a lower level of antioxidants caused a decrease in the overall AC and TPC in fortified chocolate products.

The prepared DCh without and with plant extracts were exposed to a three-phased in vitro static digestion process simulating oral, gastric, and intestinal circumstances. The effect of simulated digestion on AC and TPC in all chocolates was estimated and presented in Table 3. As can be seen, TPC in DCh samples without and with EFrE and EFlE, DPPH of DCh + EFrE, and ABTS of DCh containing only ZnL did not change significantly after in vitro digestion (Duncan test, Table 3). This suggests that the studied chocolates might be a great source of bioaccessible phenolic compounds.

However, the reducing potencies of all studied physiological extracts determined by CUPRAC and FRAP assays were about 2–5 times lower than undigested samples. Interestingly, radical scavenging activity of enriched chocolates analyzed by the ABTS test decreased by 30%–79% after digestion, whereas this physiological process caused lower losses (20–58%) of total bioactive compounds present in DCh, DCh + EFlE and DCh + ChFrE, which were capable of scavenging the DPPH radical (Table 3). These discrepancies between AC values may be caused by differences in the matrix composition of the digested chocolates (without and with plant extracts), which contributed to the gradual release of antioxidants during in vitro digestion. The decrease in reducing activity and scavenging activity of the investigated chocolates after simulated digestion may be due to the loss of the bioactive compounds and/or chemical transformations.

It is evident that the standardized static in vitro digestion of chocolates largely contributes to the structural modification and antioxidative activity alteration of their functional components such as polyphenols, hence a change in antioxidant properties of the consumed chocolates. However, compared with the control sample of DCh containing only ZnL, a significant increase in AC and TPC in all physiological extracts of enriched chocolates was observed (Table 3, Duncan test).

Additionally, Martini et al. [6] found that in vitro gastrointestinal digestion processes decreased the antioxidant properties of DCh without and with Sakura green tea and turmeric powder when analyzed by ABTS, FRAP, and F–C (Table 4). In contrast, DPPH and TPC values of compound chocolate samples fortified with black carrot extract significantly increased after in vitro digestion (Table 4, [12]). Thus, these supplemented confectionery products were advantageous for delivering and transporting phenolic and other antioxidant compounds.

### 2.3. Physicochemical Parameters of Chocolates

The moisture content (MC) of plain chocolate is related to the sequence of processes that uses a thermal treatment. Moisture is primarily decreased in the conching process. Therefore, the MC is a criterion for terminating this process [23]. In addition, water amount in chocolates contributed to their flow and sensory properties: color, appearance (mainly sugar bloom), grittiness, and hardness.

It can be noted that humidity of DCh containing ZnL without plant additives was significantly lower (MC = 0.30%) than DCh with plant extracts (MC = 0.37–0.73%) (Table 5). 

This can be explained by the presence of moisture in added plant powder extracts, whereas ZnL was the ingredient of DCh with the lowest initial MC. Although, the ZnL amount (0.0065%) in the prepared chocolates was 3 orders less than the concentration of plant extracts (5%). The moisture levels in the ingredients used and the processing method applied affected the final MC observed in chocolates. On the other hand, the water amount in chocolate formulations can be attributed to the hygroscopicity of additives. For this reason, ChFrE had relatively lower MC and hygroscopic properties than EFrE and EFlE. A low water binding capacity of ChFrE added to DCh led to moisture reduction during the conching process. Hence, an approximately 2 times lower moisture uptake was observed for DCh + ChFrE compared with MC in DCh + EFrE and DCh + EFlE (Table 5).

For comparison, DCh enriched with yellow tea extract had a higher MC (1.56 g/100 g) than the control sample (MC = 1.32 g/100 g) [7]. In contrast, the MC (1.78%) insignificantly decreased with increasing cinnamon bark oleoresin microcapsule content (c = 4, 6 and 8%) added to DCh bars (MC = 1.55–1.68%) [24].

Moreover, the viscosity of DCh (2979.24 mPa⋅s) increased after the incorporation of plant extracts (3239.72–4509.56 mPa⋅s) because moisture level has a severe thickening effect on chocolate. In the presence of water on the surface of the sugar particles, they start sticking together and form agglomerates, impeding the flow.

On the other hand, the increase in viscosity values for enriched chocolates was most likely to occur due to a decrease in the fat content (FC) in these samples (Table 5). Therefore, the plain chocolate without additives revealed the lowest MC and viscosity value, which was compensated by the highest free fat phase content.

Similarly, the addition of 2% yellow tea powdered extract caused a reduction in the fat phase in DCh (FC = 28.53 and 27.98 g/100 g for control and enriched chocolate samples, respectively) [7].

Insignificant differences in viscosity results for DCh + EFrE and DCh + ChFrE were likely caused by similar pectin content in these additives (Duncan test, Table 5). It is well known that pectins undergo gelatination during the heating process, and that the viscosity of enriched chocolate products increases.

The plastic viscosity of DCh (1.58 Pa⋅s) also increased (1.82–2.31 Pa⋅s) with an increase in concentrated raspberry leaf extract amounts in a sample from 1 to 3% [5].

### 2.4. Microstructural Properties of Chocolates

**Figure 1:**
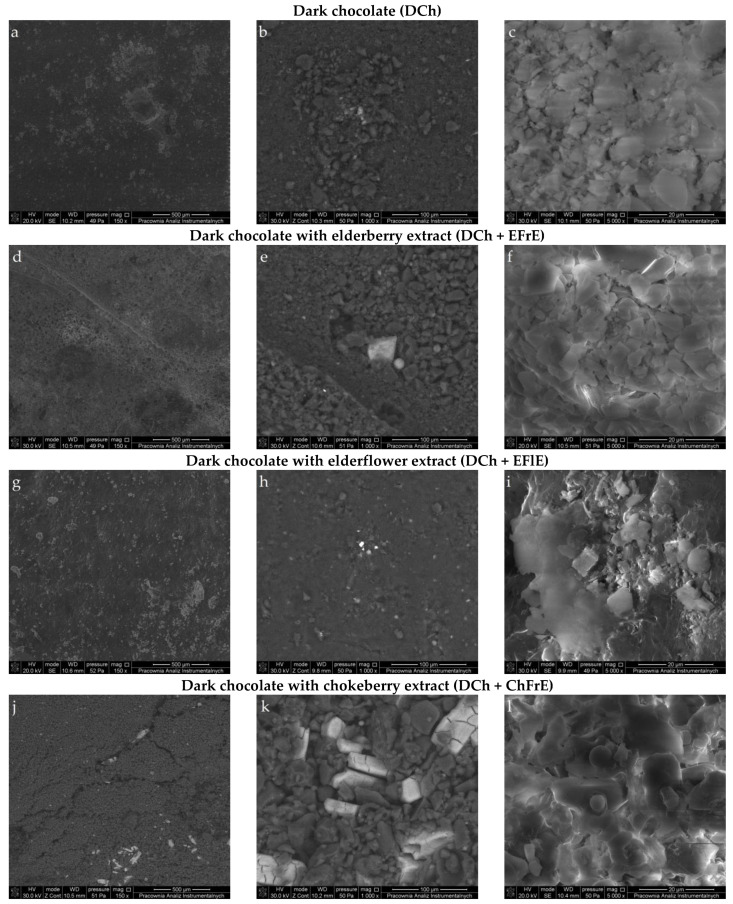
Scanning electron micrographs of dark chocolate containing ZnL (**a**,**b**,**c**), chocolate with EFrE (**d**,**e**,**f**), chocolate with EFlE (**g**,**h**,**i**), and chocolate with ChFrE (**j**,**k**,**l**) at 150, 1000 and 5000× magnification, respectively.

The SEM was used to image the effect of added fruits and flower extract powders on the surface morphology of DCh. The microstructural analysis of the chocolates without and with plant extracts revealed clear variations in crystalline network structure, inter-crystal connections, and particle distribution using 150×, 1000×, and 5000× magnification (Figure 1). The surface morphology of the chocolates was characterized by rough, sharp texture, and flaky surfaces formed by crystallized cocoa butter as the suspending medium. SEM pictures of all investigated DCh samples showed a heterogeneous and dry system made of clusters of crystals and agglomerated large structures with three-dimensional surfaces and irregular cavities. These large structures were made of sugar and cocoa crystals having irregular forms and sizes at the mass surface. The added ZnL can also be seen along with the cocoa mass as particles smaller than cocoa and sugar particles that adhered to the larger and coarser cocoa particles. Therefore, some agglomerates with relatively small edges were found in control chocolate without plant extracts (Figure 1 a–c), whereas a rough structure with relatively sharp edges can be observed in the fortified chocolate samples containing active compounds (Figure 1 d–l). This porous structure of DCh was the location for the entrapment of bioactive compounds present in added plant extracts.

On the other hand, the chocolate with plant extracts contained higher humidity (Table 5) and could induce more amorphous sugar formation during chocolate production. Moreover, fortified chocolates showed a surface that was partly covered by protrusions and pores (Figure 1 d–l). It is noteworthy that small particles filled the voids between big particles, which might increase packing fraction.

However, smaller particles of yoghurt powder added to probiotic milk chocolate in a concentration of 50% adhered to the larger and coarser cocoa and sugar particles. The cocoa particles were completely covered by smaller yoghurt powder particles in chocolate fortified with 100% yoghurt powder [25].

### 2.5. Energy Spectra Provided by SEM/EDS

**Figure 2:**
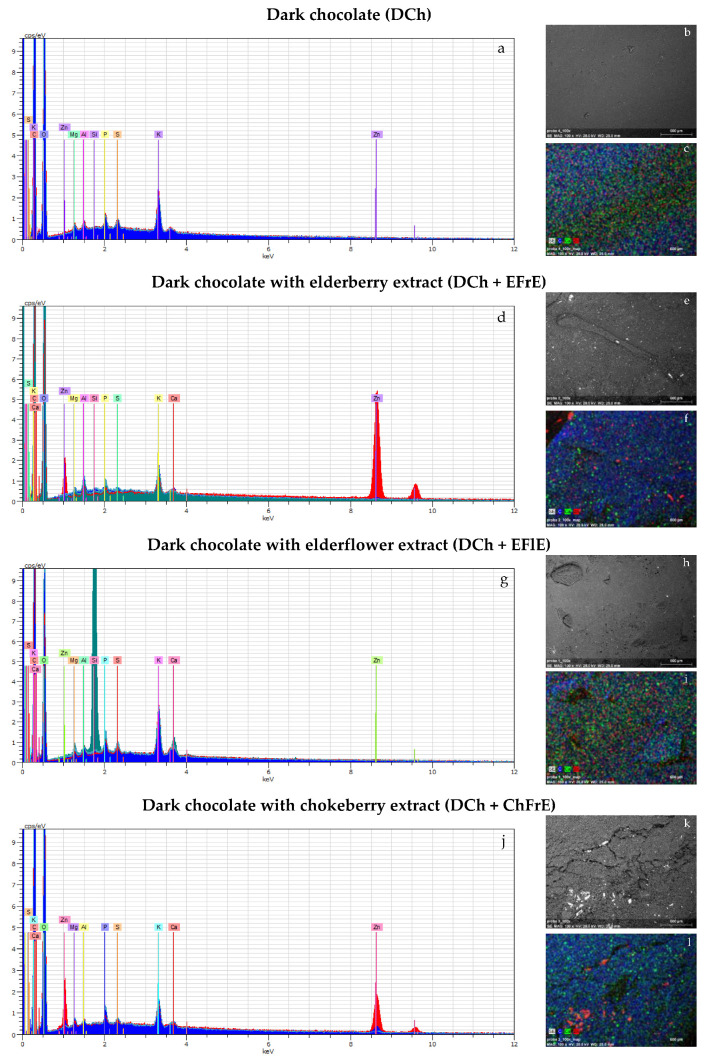
EDS spectra (**a**,**d**,**g**,**j**), SEM images (**b**,**e**,**h**,**k**) and mapping images by SEM–EDS (**c**,**f**,**i**,**l**) of dark chocolates without and with EFrE, EFlE and ChFrE.

The presence of chemical elements in the prepared chocolates and their relative compositions can also be determined using dispersive energy spectroscopy coupled with the SEM instrument (Figure 2).

EDS analysis through SEM confirmed the presence of Zn in all samples as indicated by peaks in EDS graphs (Figure 2a,d,g,j), but its concentration in enriched chocolates varied from 0.15% to 13.53%. This can be explained the possibility that Zn ion sorption had occurred on the surface of added plant extracts, and Zn aggregates were created. In contrast, an even distribution of Zn ions in the lowest amount (0.14–0.16%) was found for chocolate without plant extracts (Figure 2a–c).

The presence of carbon and oxygen can be attributed to the main organic ingredients of chocolates, such as cocoa butter and sugar. The same amounts of cocoa butter and sugar were used during the preparation of each chocolate. Thus, all EDS images contained carbon and oxygen at similar levels (C = 16.94–20.66%, O = 68.20–79.65%). High oxygen content on the surface of studied chocolates was associated with reactive oxygen-containing functional groups (carbonyl, hydroxyl, etc.).

Surprisingly, the atomic percentages of carbon (78.47–81.51%) and oxygen (18.49–21.53%) in the selected areas of untempered and bloomed chocolate samples with 38% cocoa butter estimated by SEM-EDS were exactly the opposite [26]. However, it is well known that EDS quantitative analysis of light elements may be considered only an estimate due to the intense absorption of X-ray emission inside the sample and the minimal excitation energy of the light elements (max. 1 keV) [27].

Moreover, signals from phosphorus, magnesium, and potassium were identified in the EDS spectra of analyzed chocolates (Figure 2a,d,g,j). This can be explained by the fact that cacao particles contain these elements [28]. However, the localization of phosphorus, magnesium, potassium, calcium, and sulfur indicated that they can also come from the added plant extracts. Interestingly, amongst these inorganic elements, K (0.53–1.64%) was the most abundant in all samples, and only DCh + EFlE had 2 times higher content of Ca (0.46%) than Mg (0.20%). The Ca and Mg levels in the remaining chocolates ranged between 0.12–0.19% and 0.28–0.46%, respectively. However, the appearance of Al in the spectra could be due to the aluminum stub on which adhesive tape with the powder sample was adhered.

## 3. Materials and Methods

### 3.1. Chemicals

All reagents were of analytical or HPLC grade and were purchased from Merck Life Science Sp. z o.o. (Poznań, Poland): 2,2-diphenyl-1-picrylhydrazyl radical (DPPH), 2,2′-azino-bis(3-ethylbenzothiazoline-6-sulphonic acid) diammonium salt (ABTS), 2,4,6-tris(2-pyridyl)-s-triazine (TPTZ, 99%), neocuproine (98%), Folin–Ciocalteu’s phenol reagent (F–C reagent, 2 N), Trolox (6-hydroxy-2,5,7,8-tetramethylchromane-2-carboxylic acid) (TE, 97%), gallic acid (3,4,5-trihydroxybenzoic acid) (GA, 98%), iron(III) chloride hexahydrate, sodium acetate, hydrochloric acid, ammonium acetate, copper(II) chloride, potassium persulfate, pancreatin from porcine pancreas, α-amylase from human saliva (300–1500 units/mg), pepsin, bile salts, acetic acid, acetone, methanol (99.8%), ethanol (95.0%), sodium carbonate, and n-hexane. Redistilled water was used for preparation of solutions.

### 3.2. Materials

Plain dark chocolate (DCh) with 45% cocoa solids was produced through a classical technological process in the chocolate factory UNION CHOCOLATE Sp. z o.o. located in Żychlin (Poland).

Concentrated and vacuum-dried extracts from *Sambucus nigra* L. fruits (DER 4:1) (EFrE) and flowers (DER 4:1) (EFlE), as well as a standardized extract from *Aronia melanocarpa* (Michx.) Elliott fruits (ChFrE) separated by column chromatography were supplied by Greenvit Botanical Extracts Manufacturer in Zambrów (Poland). Zinc lactate (ZnL) (PURAMEX^®^ ZN), a nutritional additive, was obtained from Corbion Group Netherlands B.V. (Amsterdam, The Netherlands).

### 3.3. Ultrasound-Assisted Extraction of Antioxidants from Plants

Extraction of antioxidants from EFrE, EFlE, and ChFrE was performed using an ultrasonic water bath (5200DTD, Chemland, Stargard Szczeciński, Poland) at power and frequency of 180 W and 40 kHz, respectively, equipped with a digital timer and temperature controller. Exactly, 0.5 g of each plant extract was mixed with hot redistilled water (25 mL) in Erlenmeyer flasks, stirred, and placed in an ultrasonic bath. Water in the ultrasonic bath was circulated and regulated at a constant temperature (25 ± 0.3 °C) to avoid water temperature increases as a result of exposure to ultrasound. Each sample was sonicated in duplicate for 10 min and centrifuged at 1880× *g* for 15 min (centrifuge MPW-54, Chemland, Stargard Szczeciński, Poland).

### 3.4. Preparation of Chocolates Fortified with Plant Extracts and Extraction Procedure

Dark chocolates incorporated with powder extracts from elderberries (DCh + EFrE), elderflowers (DCh + EFlE), and chokeberries (DCh + ChFrE), respectively, as functional ingredients were prepared in the Confectionery Factory (Kopernik S.A., Toruń, Poland) using a tempering machine with a mold filling device (Pomati T35, Codogno LO, Italy). The following procedure was used for the supplementation of chocolates: DCh was melted at 45 °C, and after the addition of ZnL (0.0065%) and each plant extract (5%), liquid chocolate was mixed in a mixer (Pomati S150, Codogno LO, Italy) and tempered at a working temperature of 31 °C. Then, polycarbonate molds were filled with the prepared chocolates. The weight of enriched chocolates in each mold was 95 g. The obtained chocolates were cooled in a cooling tunnel (Kreüter, Kühlkanal Universal K.K. 1050, Hamburg, Germany) at 10 °C for 30 min and taken out from the molds, after which they were stored at ambient temperature until the analyses.

Chocolate extracts were prepared using a conventional extraction technique as previously described by Adamson et al. [29] with some modifications. The ground chocolate samples (5.0 g) were defatted twice with 50 mL of n-hexane at room temperature for 30 min. The remaining defatted solids were air-dried for 24 h to evaporate the residual solvent. Antioxidants were extracted from each defatted chocolate sample (2.0 g) with 10 mL of an acetone–water–acetic acid mixture (Ac:H_2_O:AA = 70:29.8:0.2, *v/v/v*) by using a shaker SHKA 2508-1CE (Labo Plus, Warszawa, Poland) for 30 min. Extractions were carried out two times at room temperature. The combined extracts were filtered using polytetrafluorethylene syringe filters (PTFE, pore size 0.20 μm/diameter 13 mm, Merck Life Science Sp. z o.o., Poznań, Poland) and stored in a refrigerator until the AC and TPC analyses.

### 3.5. In Vitro Simulated Digestion of Chocolates

An in vitro digestion analysis mimicking the physiological situation in the digestive tract (simulated salivary fluid, simulated gastric fluid, and simulated intestinal fluid) was used to evaluate the bioaccessibility of antioxidant compounds in chocolates according to the procedure described by Dala-Paula et al. [13]. The simulated digestion procedures were as follows, 3 g of each tested chocolate was mixed with 3 mL simulated saliva (phosphate buffer solution: 0.04% NaCl and 0.004% CaCl_2_, pH 6.9) containing 0.07 mg α-amylase in a centrifuge tube. Samples were subsequently incubated at 37 °C for 5 min in a shaking water bath type 357 (ELPAN, Lubawa, Poland). Then, the samples were mixed with 10 mL of simulated gastric fluid prepared by dissolving 1% pepsin, 3 g of NaCl, and 9 mL of HCl in 1 L of water (the pH was adjusted to 1.3 using 1 M HCl) and incubated at 37 °C for 120 min to simulate stomach conditions. The gastric digests were maintained on ice for 10 min to stop pepsin digestion. For the intestinal digestion stage, the pH of the gastric digests was raised to 6.5 by dropwise addition of 1 M NaHCO_3_. Then an amount of freshly prepared pancreatin–bile salt solution sufficient to provide 0.005 g pancreatin and 0.03 g bile salt/g sample was added, and incubation was continued for an additional 120 min at the same temperature. To stop intestinal digestion, the sample was kept for 10 min in an ice bath. The pH was then adjusted to 7.2 by dropwise addition of 0.5 M NaOH. All samples were filtered (nylon filters of 0.45 μm, Merck Life Science Sp. z o.o., Poznań, Poland) and stored in opaque vials at 4 °C until analysis.

### 3.6. Physicochemical Analysis of Chocolates

#### 3.6.1. Moisture and Fat Determination

The representative samples from each chocolate were analyzed for proximal composition (MC and FC) using an Instalab 600 Product Near Infrared Reflectance (NIR) Analyzer (Dickey-John Inc. Minneapolis, MI, USA). Analyzed chocolate was ground to uniform particle size (0.5 mm), then the sample cup was carefully filled with ground chocolate. Next, ground chocolate was gently tamped into the cup. The chocolate surface should be flat and flush with the rim of the cup. After closing, the sample cup was placed into the sample cell of the NIR product analyzer, and spectral data were recorded at room temperature as log(1/R), where R is the reflectance energy. Afterward, results of humidity and FC were read from the analyzer display. To minimize sampling error, triplicate samples were analyzed for all the samples of the calibration set. The average spectral data were used for NIR calibration.

#### 3.6.2. Viscosity Determination

Chocolate viscosity was measured using a rotational viscometer RN 4.1 type produced by HAAKE Medingen GmbH (Ottendorf-Okrilla, Germany). Each chocolate sample (100 g) was placed in a canister closed with a lid. The sample was heated to 50 °C and held at 50 °C. The sample was mixed with a baguette to remove lumps and air bubbles every 15 min and controlled. The analysis was performed 2 h after the start of melting the chocolate, and the means of triplicate runs were recorded.

### 3.7. Antioxidant Capacity Determination

The AC and TPC in EFrE, EFlE, ChFrE, and chocolates enriched with them were determined by spectrophotometric DPPH, ABTS, CUPRAC, FRAP, and F–C methods according to procedures described in our previous article with some minor modifications [30]. The resulting absorbance of each obtained solution was measured in five repetitions using a Hitachi U-2900 spectrophotometer (Tokyo, Japan) in a 1-cm glass cell. The AC results were expressed as μmol Trolox equivalents (TE) per 1 g of sample, while TPC values were expressed as mg gallic acid (GA) equivalents per 1 g of sample.

### 3.8. Scanning Electron Microscopy with Energy Dispersive X-ray Spectrometer

The morphology of chocolates was observed by scanning electron microscopy/focused ion beam (SEM/FIB) using Quanta 3D FEG microscope (Carl Zeiss, Göttingen, Germany). The micrographs were recorded under a low vacuum using a secondary electron detector (SE), and accelerating voltage ranging between 20.0 and 30.0 kV was chosen for SEM analysis. Samples were defatted and frozen in liquid nitrogen before measurements.

Additionally, the morphology and elemental composition of the defatted chocolates without and with plant extracts were analyzed with a scanning electron microscopy (SEM) LEO Electron Microscopy Ltd., 1430 VP (Cambridge, UK) equipped with detectors of backscattered electron (BSE), cathodoluminescence (CL), and an energy dispersive X-ray spectrometer (EDS) Quantax with an XFlash 4010 detector (Bruker AXS microanalysis GmbH, Berlin, Germany). EDS was used for the element-mapping analysis of chocolate samples. The elemental composition at different points of each sample was carried out at acceleration voltage, HV: 28.0 kV, live time 40 s, working distance, WD: 25.0 mm, and 100× magnification.

### 3.9. Statistical Analysis

The obtained results were expressed as mean ± standard deviation (SD). All data were statistically tested, and the means were compared by one-way analysis of variance (ANOVA) with subsequent comparisons by Duncan’s test at a 0.05 significance level using Statistica 8.0 software (StatSoft, Tulsa, OK, USA).

## 4. Conclusions

In this study, DCh samples containing ZnL were successfully enriched with EFrE, EFlE, and ChFrE, which affected the physicochemical, antioxidant, and microstructural properties of new confectionery products. Moreover, the digestive stability of natural antioxidants from plant extracts present in fortified chocolates was estimated using the standardized static in vitro digestion model. Enrichment of DCh with plant extracts enhanced scavenging radical activity (DPPH and ABTS values), reducing ability (CUPRAC and FRAP values), and the level of total phenolics in undigested and digested chocolates. This increase is strictly associated with the botanical origin of extracts having high antioxidant potential as analyzed by the mentioned analytical methods. Changes in the composition of DCh by the addition of ZnL and plant extracts affected the extraction and release of bioactive compounds from the chocolate matrix, which in turn had an impact on their bioaccessibility and bioavailability. The addition of plant extracts to DCh resulted in higher MC, viscosity, and/or amorphous parts creating agglomeration of solid particles. Combining SEM imaging and EDS analysis revealed that unsupplemented and supplemented chocolates had noticeable differences in chemical composition and surface characteristics such as roughness, grain size, and presence of pores or protrusions.

With further improvements in the processing technology of cocoa-based products, EFrE, EFlE, and ChFrE can serve as healthy additives to plain chocolate, resulting in products with better health benefits for health-conscious consumers and the entire consuming populace.

## Figures and Tables

**Table 1:** Antioxidant capacity and total phenolic content in plant extracts added to chocolates.

Sample	DPPH * ± SD (μmol TE/g)	ABTS * ± SD (μmol TE/g)	CUPRAC * ± SD (μmol TE/g)	FRAP * ± SD (μmol TE/g)	TPC * ± SD (mg GA/g)
EFrE	1911.6 ± 56.9 ^a^	2337.1 ± 18.9 ^a^	5710.4 ± 99.5 ^a^	229.8 ± 4.5 ^a^	32.5 ± 1.3 ^a^
EFlE	4765.0 ± 26.6 ^b^	6258.7 ± 131.2 ^b^	6224.0 ±54.5 ^b^	595.2 ± 3.4 ^b^	81.9 ± 3.9 ^b^
ChFrE	22,269.7 ± 161.4 ^c^	27,194.2 ± 242.0 ^c^	113,882.3 ± 303.1 ^c^	2341.3 ± 10.3 ^c^	3506 ± 2.3 ^c^

*n = 3; SD―standard deviation; Mean values within the same column marked by different superscript small letters (a–c) are significantly different (one-way ANOVA and Duncan test, *p* < 0.05).

**Table 2:** Antioxidant capacity and total phenolic content in elderberry, elderflower, and chokeberry extracts, as determined by other authors.

Analytical Methods	EFrE	EFlE	ChFrE
DPPH	100.16 μmol TE/g [16]		181.07 μmol TE/g [16]
	30–45% [18]		90–95% [18]
	50.25–67.69% [19]	91.95–94.15% [19]	
ABTS	37.91 μmol TE/g [16]		78.90 μmol TE/g [16]
	44.02 g/kg [17]		54.27 g/kg [17]
	3.20–36.50 mM TE/kg [20]	44.87–118.26 mM TE/kg [20]	
	397.5–581.3 μmol TE/g [21]	327.7–421.5 μmol TE/g [21]	
F–C	4415.33 mg/kg [16]		7194.40 mg/kg [16]
	80.71 g/kg [17]		115.15 g/kg [17]
	700–1050 mg/100 g [18]		3100–3600 mg GA/100 g [18]
	2687.6–6831.1 mg GA/kg [20]	7410–40,137 mg GA/kg [20]	
	5678.8–7087.3 mg ChA/100 g [21]	6164.4–7561.8 mg ChA/100 g [21]	

GA―gallic acid; ChA―chlorogenic acid; TE―Trolox

**Table 3:** Antioxidant capacity and total phenolic content in the investigated chocolates before (Ac:H_2_O:AA extract) and after (physiological extract) in vitro digestion.

Analytical Method	DCh		DCh + EFrE		DCh + EFlE		DCh + ChFrE	
	Ac:H_2_O:AA Extract	Physiological Extract	Ac:H_2_O:AA Extract	Physiological Extract	Ac:H_2_O:AA Extract	Physiological Extract	Ac:H_2_O:AA Extract	Physiological Extract
DPPH * ± SD (μmol TE/g)	144.2 ± 5.3 ^b^	72.2 ± 5.1 ^a^	149.3 ± 4.6 ^b^	146.1 ± 1.9 ^b^	364.3 ± 17.1 ^c^	151.4 ± 12.5 ^b^	942.7 ± 31.0 ^e^	745.6 ± 12.7 ^d^
ABTS * ± SD (μmol TE/g)	433.9 ± 11.5 ^a,b^	407.0 ± 1.1 ^a^	672.6 ± 2.5 ^d^	467.6 ± 1.6 ^b^	1211.0 ± 8.92 ^f^	575.8 ± 0.7 ^c^	3592.0 ± 76.7 ^g^	755.3 ± 6.4 ^e^
CUPRAC * ± SD (μmol TE/g)	2985.4 ± 14.7 ^e^	1346.3 ± 19.7 ^c^	3118.5 ± 7.8 ^f^	883.1 ± 36.0 ^a^	3752.3 ± 27.3 ^g^	1222.9 ± 46.6 ^b^	12,945.7 ± 132.2 ^h^	2773.3 ± 53.9 ^d^
FRAP * ± SD (μmol TE/g)	66.5 ± 0.7 ^d^	29.9 ± 1.3 ^a^	96.0 ± 1.1 ^e^	44.3 ± 0.3 ^b^	102.2 ± 0.8 ^f^	57.8 ± 0.9 ^c^	350.7 ± 2.3 ^h^	133.7 ± 2.0 ^g^
F–C * ± SD (mg GA/g)	11.7 ± 0.5 ^a,b^	9.8 ± 1.1 ^a^	12.8 ± 0.4 ^b^	11.1 ± 2.2 ^a,b^	17.9 ± 0.5 ^c^	17.3 ± 1.1 ^c^	70.9 ± 2.2 ^e^	67.6 ± 2.8 ^d^

*n = 3; SD―standard deviation; Mean values within the same row marked by different superscript small letters (a–h) are significantly different (one-way ANOVA and Duncan test, *p* < 0.05).

**Table 4:** Antioxidant capacity and total phenolic content in plain and enriched chocolates, as determined by other authors.

Analytical Methods	Plain Chocolates	Enriched Chocolates
Chemical extracts		
DPPH	0.044 mmol TE/g for ChP [3]	0.022–0.031 mmol TE/g for ChP + longan [3]
		0.018–0.028 mmol TE/g for ChP + lychee [3]
	4012 mg TE/100 g for DCh [7]	4373 mg TE/100 g for DCh + yellow tea extract [7]
	0.08 mg TE/g for CCh [12]	0.16–0.40 mg TE/g for CCh + black carrot extract [12]
ABTS	1.91 mmol TE/L for DCh [2]	2.04 mmol TE/L for DCh + cranberries [2]
	0.060 mmol TE/g for ChP [3]	0.022–0.044 mmol TE/g for ChP + longan [3]
		0.028–0.039 mmol TE/g for ChP + lychee [3]
	9 mmol/g for DCh [5]	9–11.5 mmol/g for DCh + red raspberry leaves extract [5]
	11 mmol TE/100 g for DCh [6]	15.4 mmol TE/100 g for DCh + Sakura green tea leaves [6]
		12.2 mmol TE/100 g for DCh + turmeric powder [6]
	285 mg TE/100 g for DCh [7]	386 mg TE/100 g for DCh + yellow tea extract [7]
FRAP	8.06 mmol Fe(II)/L for DCh [2]	9.20 mmol Fe(II)/L for DCh + cranberries [2]
	13 mmol/g for DCh [5]	13–14.5 mmol/g for DCh + red raspberry leaves extract [5]
	10.1 mmol TE/100 g for DCh [6]	15.4 mmol TE/100 g for DCh + Sakura green tea leaves [6]
		10.3 mmol TE/100 g for DCh + turmeric powder [6]
F–C	4.8 mg GA/g for DCh [2]	6.2 mg GA/g for DCh + prunes [2]
	10 mg GA/g for ChP [3]	5–7 mg GA/g for ChP + longan [3]
		4.2–6.1 mg GA/g for ChP + lychee [3]
	15,425 μmol GA/100 g for DCh [6]	20,090 μmol GA/100 g for DCh + Sakura green tea leaves [6]
		17,887 μmol GA/100 g for DCh + turmeric powder [6]
	1760 mg C/100 g for DCh [7]	2400 mg C/100 g for DCh + yellow tea extract [7]
	56.0 mg GA/kg for CCh [12]	85.0–117.7 mg GA/kg for CCh + black carrot extract [12]
Physiological extracts		
DPPH	0.12 mg TE/g for CCh [12]	0.25–0.56 mg TE/g for CCh + black carrot extract [12]
ABTS	1.8–10 mmol TE/100 g for DCh [6]	2.2–11.4 mmol TE/100 g for DCh + Sakura green tea leaves [6]
		2.1–9.9 mmol TE/100 g for DCh + turmeric powder [6]
FRAP	0.9–3.9 mmol TE/100 g for DCh [6]	1–5.4 mmol TE/100 g for DCh + Sakura green tea leaves [6]
		0.8–4 mmol TE/100 g for DCh + turmeric powder [6]
F–C	1800–10,100 μmol GA/100 g for DCh [6]	1850–13,900 μmol GA/100 g for DCh + Sakura green tea leaves [6]
		1900–7800 μmol GA/100 g for DCh + turmeric powder [6]
	70.8 mg GA/kg for CCh [12]	106.6–287.7 mg GA/kg for CCh + black carrot extract [12]

DCh―dark chocolate; ChP―chocolate pralines; CCh―compound chocolate; C―catechin equivalent; TE―Trolox.

**Table 5:** Physicochemical parameters of the investigated chocolates.

Sample	Moisture Content ± SD (%)	Fat content ± SD (%)	Viscosity ± SD (mPa⋅s)
DCh	0.30 ± 0.01 ^a^	32.91 ± 0.35 ^d^	2979.24 ± 48.84 ^a^
DCh + EFrE	0.73 ± 0.02 ^d^	29.69 ± 0.28 ^b^	4493.28 ± 48.94 ^c^
DCh + EFlE	0.65 ± 0.02 ^c^	30.78 ± 0.15 ^c^	3239.72 ± 28.20 ^b^
DCh + ChFrE	0.37 ± 0.02 ^b^	29.12 ± 0.22 ^a^	4509.56 ± 28.19 ^c^

*n = 3; SD―standard deviation; Mean values within the same column are marked by different superscript small letters (a–d) are significantly different (one-way ANOVA and Duncan test, *p* < 0.05).

## Data Availability

The data presented in this study are available on request from the corresponding author.

## Sample Availability

Samples of the compounds are available from the authors.
